# Evolving Institutional Arrangements for use of an ecosystem approach in restoring Great Lakes Areas of Concern

**DOI:** 10.3390/su13031532

**Published:** 2021-02-01

**Authors:** Peter J. Alsip, John H. Hartig, Gail Krantzberg, Kathleen C. Williams, Julia Wondolleck

**Affiliations:** 1 Cooperative Institute for Great Lakes Research, University of Michigan, 4840 South State Road, Ann Arbor, Michigan 48108; 2 Great Lakes Institute for Environmental Research, University of Windsor, 2900 Riverside Dr. West, Windsor, Ontario N9C 1A2 Canada; 3 Booth School of Engineering Practice and Technology, McMaster University, 1280 Main Street West, Engineering Technology Building, Hamilton, Ontario L8S 0A3 Canada; 4 Great Lakes Toxicology and Ecology Division, U.S. Environmental Protection Agency, 6201 Congdon Blvd., Duluth, MN 55804; 5 University of Michigan, School for Environment and Sustainability, 440 Church St., Ann Arbor, MI 48109

**Keywords:** Ecosystem-based management, Institutional Frameworks, Water Governance, Remedial Action Plans

## Abstract

The 1987 Canada-U.S. Great Lakes Water Quality Agreement required Remedial Action Plans (RAPs) be collaboratively generated between local stakeholders and government agencies to implement an ecosystem approach in cleaning up 43 historically polluted Areas of Concern (AOCs) throughout the Laurentian Great Lakes. The institutional arrangements that have emerged over the past 35 years to foster an ecosystem approach in RAPs are expected to have changed over time and be varied in some aspects—reflecting unique socio-ecological contexts of each AOC—while also sharing some characteristics that were either derived from the minimally prescribed framework or developed convergently. Here we surveyed institutional arrangements to describe changes over time relevant to advancing an ecosystem approach in restoring beneficial uses in the 43 AOCs. While eight AOCs evidenced little institutional change, the remaining 35 AOCs demonstrated a growing involvement of local organizations in RAPs, which has enhanced local capacity and ownership and helped strengthen connections to broader watershed initiatives. We also noted an expansion of strategic partnerships that has strengthened science-policy-management linkages and an increasing emphasis on sustainability among RAP institutions. Our study details how institutional arrangements in a decentralized restoration program have evolved to implement an ecosystem approach and address new challenges

## 1. Introduction

An ecosystem approach is a socio-ecological style of resource conservation that accounts for the interrelationships among land, air, water, and all living things, including humans, and involves all user or stakeholder groups in comprehensive management and collaborative governance [1–3]. Collaborative governance is defined as the processes and structures of public policy decision-making and management that engage people constructively across the boundaries of public agencies, levels of government, and/or the public, private and civic spheres in order to carry out a public purpose that could not otherwise be accomplished [4]. While an ecosystem approach is considered an innovative and integrative management method for protecting, restoring, and improving the health of large ecosystems, applying it to ecosystems spanning multiple jurisdictions can be particularly challenging [5–9]. In this study, we seek to understand how institutional structures have evolved to adopt and promote the use of an ecosystem approach in a non-regulatory restoration program in the Laurentian Great Lakes.

**Figure 1.**
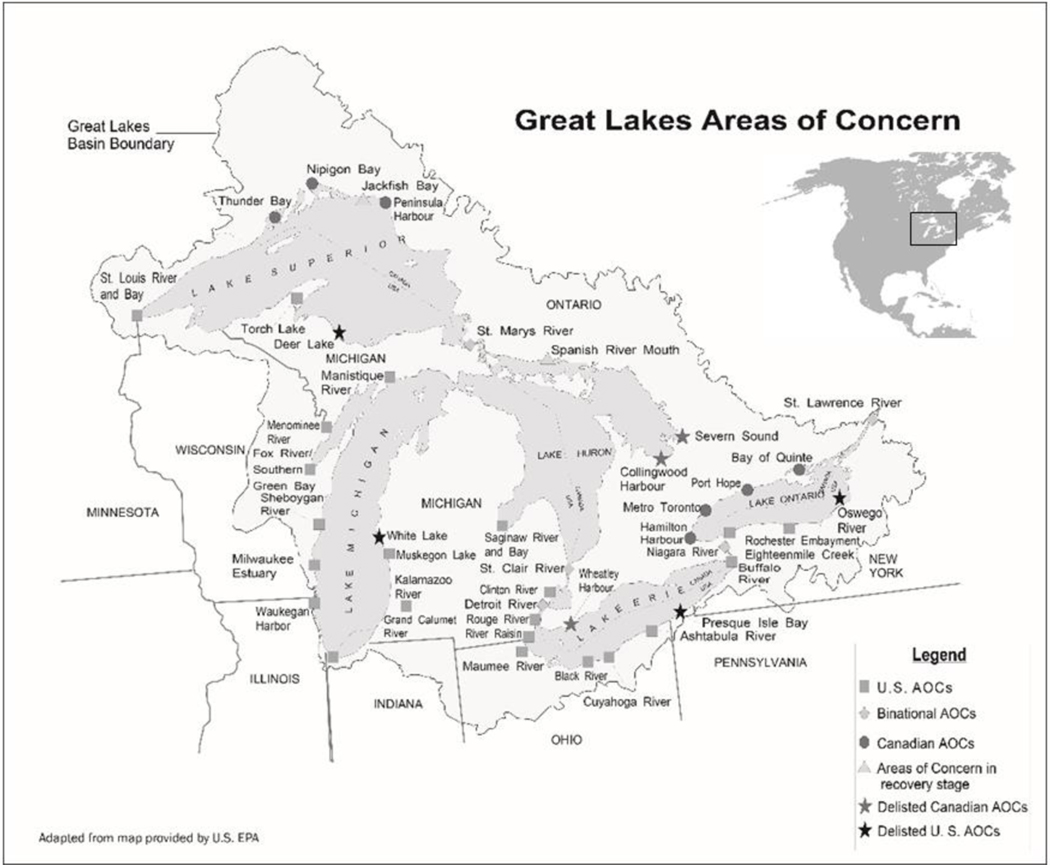
The 43 Areas of Concern (AOCs) identified in the Laurentian Great Lakes Basin of North America. United States’ AOCs are represented by squares, Canadian AOCs by circles, and Binational AOCs by diamonds. AOCs in the recovery stage (i.e. all remedial actions completed and monitoring of beneficial use restoration is underway) are indicated by a triangle and delisted AOCs are represented as stars (US: Black; CAN: Grey).

In 1985, an ecosystem-based model of management was introduced into the Laurentian Great Lakes under the auspices of the Canada-U.S. Great Lakes Water Quality Agreement (GLWQA). This agreement requires that collaboratively generated Remedial Action Plans (RAPs) in 43 historically polluted Areas of Concern (AOCs) (Figure 1) be developed and implemented using an ecosystem approach to restore impaired human and non-human Great Lakes uses, which are formally referred to as beneficial uses [3,10]. Annex 1 in the GLWQA outlines the 14 different beneficial use impairments (BUIs), which are caused by reductions in the chemical, physical, or biological integrity of the waters of the Great Lakes [3,10].

Over time, AOC restoration efforts have benefitted from several important sources of funding that demonstrated and required public and political support given the non-regulatory nature of RAPs. In Canada, early financial support was provided through the Great Lakes Cleanup Fund in the late 1980s and then replaced by the Great Lakes Sustainability Fund in 2000, which was later replaced by the Great Lakes Protection Initiative in 2017. Additionally, the Canada Ontario Agreement Respecting the Great Lakes Water Quality and Ecosystem Health has been an important source of Canadian funds since its inception in 1971 to present day. The establishment of the Great Lakes Legacy Act in 2002 was the first major source of US public funds to support restoration. This was followed by the establishment of Great Lakes Restoration Initiative in 2010, which has received $3.48 billion USD between 2010–2019 with more than a third of those funds distributed to the “Toxic Substances and AOC” focus area (https://www.glri.us/funding). RAP-related expenditures from several different funding sources in both countries totaled $22.78 billion USD countries between 1985–2019 [11]. The financial support generated to support RAPs is, in and of itself, a success and has been important to sustaining progress in AOC restoration.

The incorporation of RAPs into the 1987 Protocol to the GLWQA gave the program legitimacy [2] and represented a shift in the governance paradigm that promoted shared decision-making among stakeholders through local public advisory councils (PACs) and/or other institutional arrangements. Institutional arrangements refer to the organizational structures and mechanisms to achieve cooperative and coordinated planning and action among different organizations whose missions impact or are impacted by uses in AOCs [6,12,13]. While the most basic objective of RAPs is to shed the AOC designation (i.e., delist) by remediation and restoration of all known BUIs, the RAP process also aims to promote collaborative governance that emphasizes stakeholder empowerment [12,13]. Collaborative governance provides an interesting template for studying non-regulatory restoration and revitalization initiatives implemented in a largely decentralized manner (i.e. planning, implementation, and coordination mostly occurs at the sub-federal and local level across multiple jurisdictions instead of being led by a single authoritative entity). While RAPs are intended to be unique, place-based approaches, collaboration among all levels of government and stakeholder groups is essential for collective success around a common goal [14–16]. However, the success of RAPs in adopting and implementing an ecosystem approach has been variable and may reflect differences in local context, which can affect the degree and value of public involvement [17]. Variability in use of an ecosystem approach might also be explained through the diffusion of innovation theoretical framework, where successful RAPs involved organizations that saw the potential of the program and made it work in their AOC [15]. Kellogg [15] highlights that while the role of local stakeholder groups may differ across AOCs, they are often critical for shaping the process of lead governmental agencies in adopting an ecosystem approach. Therefore, the varied level and nature of stakeholder involvement across different AOCs would plausibly produce variability in how the locally defined approach is implemented.

The 35-year experience with RAPs provides an opportunity to investigate how local adaptation of a common management strategy unfolds in a large, institutionally rich region like the Great Lakes. In 1987, the GLWQA specifically called for broad-based public participation (i.e., the democratic way for governments and nongovernmental groups to receive public input that facilitates the identification of community goals and enables plan-makers to develop and implement appropriate plans) in RAPs without outlining clear methods for engagement. The vagueness of this mandate has encouraged locally designed ecosystem approaches to fit the unique biophysical, socioeconomic, and political contexts of each AOC. As a result, it is expected that the modern-day institutional arrangements around RAPs exhibit differences driven by local factors while maintaining certain commonalities either derived from the original institutional framework or perhaps developed convergently. However, the degree of difference in how institutional arrangements have evolved to incorporate and implement an ecosystem approach in RAPs has not been characterized but could provide useful insights into common factors of successful RAP institutional structures [18]. Moreover, understanding the role institutional arrangements play in driving and sustaining action from non-regulatory environmental policy has implications for designing and implementing other integrated resource management initiatives in the Great Lakes and elsewhere in the world [19].

The purpose of this study is to assess how RAP institutional arrangements have changed over time to apply an ecosystem approach in restoring uses and to address new and ongoing needs in each AOC. We surveyed the institutional arrangements specific to each AOC to describe changes over time and documented important changes relevant to the successful use of an ecosystem approach in restoring beneficial uses in the 43 AOCs (38 AOCs solely in either the United States or Canada; five binational AOCs, including three binational AOCs with two separate RAP processes).

## 2. Materials and Methods

We conducted a content analysis using online sources, including RAP documents, websites, or other types of media generated by involved organizations to assess changes in institutional structures since RAP initiation in 1985. RAP documents were the starting point for identifying how RAP institutional arrangements have changed over time. These documents were found at AOC’s respective federal (i.e. https://www.epa.gov/great-lakes-aocs or https://www.canada.ca/en/environment-climate-change/services/great-lakes-protection/areas-concern.html) and state/provincial agency’s websites or from the PAC’s website, if one existed. In some cases, peer-reviewed and grey literature (e.g., technical reports or theses and dissertations) were reviewed, if pertinent, to characterize RAP institutional structures and their evolution. When publicly available information was lacking, we corresponded with people involved in RAP institutional arrangements for more information.

Our analysis focused on the structural and functional components of institutional arrangements and their evolution. We employed an analytical framework that was comprised of a list of questions that guided our survey and a coding framework to further guide and interpret the data (Table 1). Our list of questions aimed at eliciting descriptive characteristics of the structure of institutional arrangements and their change over time including:

- How many AOC institutional structures changed over time and how?
- Who are the typical actors involved in RAPs and what are their roles? How has this changed over time?
- Has the RAP process led to the emergence of new institutions with foci specific or tangential to the RAP?
- From 1985 to present, how many AOCs have established a nonprofit organization?
- How many AOC institutional structures reference life after delisting or sustainability?
- How were pre-existing institutions modified to accommodate the RAP and stakeholder needs?
- How have the roles of institutions and relation among institutions changed over time to address new challenges and needs?
- Does the AOC fall within the jurisdiction of any regulatory clean-up programs (e.g., Superfund, enforcement actions, Resource Conservation and Recovery Act) and, if so, how has this affected RAP institutional structure and function?

Our coding framework is based on eight key structural/functional characteristics associated with successful stakeholder involvement and use of an ecosystem approach in RAPs (Table 1). These key characteristics summarize the organizational and process qualities necessary for long-term success of RAPs and were developed from a review of RAP literature [12,20] and other ecosystem-based work (e.g., [21]). These eight characteristics were used to help evaluate data from surveyed documents in the context of use of an ecosystem approach in RAPs and restoration of beneficial uses as called for in the GLWQA. We also documented any institutional changes that did not conform to this analytical framework but were important to understanding the evolution of a given institutional arrangement. Summaries of institutional arrangements and how they evolved are reported in Table 2.

## 3. Results

Four notable institutional adaptations were apparent from our survey. While eight AOCs evidenced little institutional change, the remaining 35 AOCs demonstrated a growing involvement of local organizations in RAPs, new mechanisms connecting RAPs to their watersheds, an expansion of strategic partnerships, and an increasing focus on the future (Table 3).

### 3.1. Growing involvement of local organizations in RAPs

Institutional arrangements in 35 of the 43 AOCs evolved over time into structures with broad stakeholder representation and active public participation. In most AOCs, organizational mechanisms for public engagement in AOC restoration were initiated by the formation of PACs. However, RAPs were bolstered in some AOCs by pre-existing local institutions or management frameworks at the outset of the program that had the capacity, understanding, and motivation to support the RAP, e.g. Rochester Embayment and Clinton River (Table 2). Over time, stakeholder representation and involvement of local organizations was broadened in most RAPs. Our survey indicates arrangements that were most evolved often included an integrative institution that was typically a nongovernmental organization (NGO), with exception to a few RAPs where municipal and county level governments played pivotal roles, e.g. Rochester Embayment’s Water Quality Management Agency (Table 2). Integrative organizations have enhanced public involvement and often were nonprofit organizations in the United States or Conservation Authorities in Canada. In our survey we also found that 32 of the 43 AOCs have established nonprofit organizations or Conservation Authorities to help build capacity, implement management actions, promote public education, and cultivate environmental stewardship. These organizations often fill many roles (e.g., public education and outreach, goal setting, research and monitoring, engaging local officials) and address many of the critical elements required of RAP institutional arrangements for implementing ecosystem-based management. The establishment and growth of these organizations has helped build local capacity and ownership, broadened the timeline of thinking to consider life after delisting, enabled a watershed focus, and, in several cases, strengthened the connection of science, policy, and management (Tables 2 & 3).

### 3.2. Enhanced institutional mechanisms for connecting RAPs to their watersheds

Institutional arrangements have also become increasingly more connected to external networks and organizations working within the same watershed (Tables 2 & 3). Organizations involved in the RAP often served as the mechanism connecting their mission of use restoration to the management of the encompassing watershed. For instance, the expansion of the organization Friends of the Buffalo River into the present-day group the Buffalo-Niagara Waterkeeper helped integrate the Niagara River RAP into restoration efforts elsewhere in the watershed (Table 2) [26]. Our survey found that in some AOCs it was sometimes deemed most efficient and effective to solve use impairments on a smaller geographic scale and work in and through a broader institutional framework to achieve watershed goals. Some of these broader institutional frameworks were RAP-centric (i.e. they originated from the RAP process with the purpose of supporting the RAP) or were centered around aspects of watershed management not derived from the RAP. For example, the Partnership for the Saginaw Bay Watershed PAC, which merged the Saginaw Basin Alliance and the Saginaw Bay Watershed Council, has voting representatives from each of its subwatersheds and also works closely with The Saginaw Bay Watershed Initiative Network to solve problems on a watershed scale (example of RAP-centric watershed framework) [27]. Contrasting this are non-RAP-centric frameworks such as in the Rochester Embayment AOC, where the Monroe County Water Quality Management Advisory Committee serves as the RAP institutional structure and coordinates with the Water Resources Board of the Finger Lakes – Lake Ontario Watershed Protection Alliance to coordinate efforts on a watershed scale (Tables 2 & 3) [28,29]. In Ontario, the four north shore Lake Superior PACs have evolved from a government-led initiative to ones that are now led by Lakehead University with a partnership with EcoSuperior, a nonprofit organization promoting stewardship in the Lake Superior basin (Table 2).

### 3.3. Expansion of strategic partnerships

Most institutional arrangements that have advanced RAP progress have created and expanded strategic partnerships among stakeholders. Common partnerships within the RAP institutional arrangement include interagency partnerships, partnerships between and among NGOs and PACs, public-private partnerships, and partnerships with universities. Partnerships were strategic in that they specifically enhanced the RAP by pooling resources, strengthening extension and outreach efforts, and/or increasing research and monitoring capacity. In several AOCs, partnerships with NGOs were initiated early on to help PACs with administrative, fiduciary, and/or technical support, and later often evolved to emphasize public outreach and education efforts, e.g. Friends of the St. Clair River (Table 2). Partnering with universities was documented in many AOCs as well, including Hamilton Harbour with McMaster University and the Wisconsin AOCs in which University of Wisconsin-Extension helped facilitate the process and contribute to public outreach and education efforts. University involvement in AOCs typically was either in public outreach and education, research and monitoring, or centralizing information. Interagency partnerships and public-private partnerships helped leverage expertise from different agencies and stakeholders while also pooling technical and financial resources, e.g. Ashtabula River Partnership (Table 2). In some AOCs, nonprofit organizations were recruited to help revitalize the RAPs and lead restoration efforts, e.g. Buffalo Niagara Waterkeeper for Buffalo River and Friends of the Detroit River for the U.S. portion of the Detroit River (Table 2).

### 3.4. Increasing focus on the future

We found that 30 of the 43 AOCs now recognize “life after delisting” or “sustainability” as part of their focus. In these AOCs, organizations have typically become the institutional mechanism for ensuring continued use of an ecosystem approach. For example, the Environmental Network of Collingwood remains active in promoting sustainability 26 years after delisting Collingwood Harbour as an AOC. In some AOCs, sustainability is being promoted through several avenues. Such is the case in the former White Lake AOC, where the Muskegon Conservation District has established an environmental stewardship program while the former PAC has reorganized into what is now called the White Lake Environmental Network through which they focus on sustaining momentum into revitalization (Table 2). The increasing prevalence of sustainability-driven organizations and programs suggests that institutional arrangements are evolving beyond the original intended scope of the AOC program (i.e., delisting) and that the program has become more effective over time through strategic partnerships that focus on stewardship and sustainability.

## 4. Discussion

The changes to institutional arrangements summarized above provide useful lessons for understanding how institutional structures can promote the use of an ecosystem approach in RAPs. The growing presence of integrative organizations has increased public participation, local ownership and capacity, and has enhanced connections between the RAP and their watersheds. Strategic collaborations and partnerships have strengthened science-policy-management linkages giving RAP institutions a greater adaptive capacity in addressing new and ongoing challenges. Additionally, sustainability has emerged as an objective, which highlights how building community support can foster a stewardship ethic that is necessary for advancing and preserving progress in remediation, restoration, and revitalization of the waterway. Our study also indicates that static institutional arrangements can be explained by the strength of their original structures and their unique regulatory contexts.

### 4.1. Growing presence of integrative organizations

#### 4.1.1. Integrative organizations increase public participation

The growing roles of local organizations in RAPs noted in our survey indicate that many institutional arrangements have evolved into structures with broad stakeholder representation and active public participation. The importance of strong local involvement in the RAP was well characterized by Krantzberg and Rich [31] who explain how an explicit focus on developing an inclusive process helped nurture local capacity in Collingwood Harbour. They demonstrate how true public participation, as opposed to mere consultation, can legitimize RAPs by supporting participatory decision-making and capacity building that is beneficial to sustaining progress throughout remediation and beyond delisting. However, recruiting and sustaining active stakeholder engagement is a challenge for many AOCs [14]. Williams (2015) [32] identified three components to effective participation in RAPs: 1) the state/province needs to create the opportunity for a local RAP organization to contribute; 2) there should be a meaningful way for citizens or advisory councils to contribute; and 3) there should be opportunities for mutual learning. Our results suggest that opportunities for public participation have been enhanced and capitalized on by local AOC actors as institutional arrangements have evolved.

We found that strong public participation was often led by local organizations that helped creatively connect the public to the RAP process. These were typically NGOs, although in some AOCs this role was attributed to local government, e.g. Rochester Embayment (Table 2), or distributed among multiple organizations, e.g. Rouge River (Table 2). We refer to these organizations as ‘integrative’ because they assimilate the perspectives of stakeholders and the public into the RAP process, they connect the RAP to other environmental and economic programs locally and regionally, and they incorporate the RAP’s goal of restoration into their own organizational objectives or mission. Additionally, these organizations were sometimes convening bodies that helped coordinate multiple agencies in RAP implementation, such as the St. Louis River Alliance for St. Louis River AOC and the Clinton River Watershed Council for the Clinton River AOC (Table 2). The increasing involvement of organizations like these is evidenced by the fact that 32 of 43 AOCs have established nonprofit organizations or worked closely through Conservation Authorities, which demonstrates a notable increase from the 14 AOCs with active nonprofits reported by Hartig and Law [12] more than 25 years ago. Thus, the growing presence of integrative organizations that rallies the community around the RAP is not only characteristic of the evolution of institutional arrangements, but also may be indicative of an empowered arrangement where the locus of environmental governance and stewardship is becoming increasingly local.

#### 4.1.2. Increasing public involvement and the resulting increase of local ownership and capacity

The growing roles of local organizations might reflect a growing sense of ownership and empowerment among local entities and stakeholders involved in the restoration and revitalization process. Local ownership can help sustain the recovery of beneficial uses thereby helping to ensure the societal, economic, and environmental sustainability of the local community [33]. From the outset of RAPs in 1985, it was recognized that these processes would not be successful if they were implemented in a top-down, command-and-control fashion, rather they would need to be undertaken in a bottom-up collaborative fashion that achieves local ownership [1]. Increasing stakeholder involvement in RAPs has cultivated local ownership and built the capacity of local governmental and nongovernmental organizations to assess issues and opportunities, creatively solve problems, acquire necessary resources, and implement remedial and preventive actions [12,33–35]. Since the beginning of the RAP program in 1985, three AOCs have stood out for immediately recognizing the importance of local ownership to solve problems and developing a locally designed ecosystem approach to restore impaired beneficial uses: Fox River/Green Bay, Hamilton Harbour, and Rouge River [1] (Table 2). In another example, Zeemering (2018) [34] demonstrated how Chippewa County Health Department, which has a representative in the St. Mary’s River AOC’s Binational PAC, became more involved over time with water quality monitoring that helped leverage relationships within and outside of the AOC program’s network to improve monitoring. This helped justify future funding for remediation—demonstrating how capacity building through engaging local stakeholders can foster local ownership that improves success of restoration efforts. The growing roles of local organizations noted in our survey not only support a general trend of increasing local ownership across AOCs documented in the literature, but it also signifies a growing capacity for more sustainable management decisions that can help maintain momentum through delisting and transition into a future with less government involvement typical of the past 35 years.

#### 4.1.3. Organizations as institutional mechanisms for connecting RAPs to their watersheds

It is generally accepted that a watershed is the appropriate unit for water resource planning and management [36], and our survey noted how many RAP institutional arrangements have evolved to enhance collaboration throughout watersheds and with multiple jurisdictions to restore beneficial uses. As documented in Tables 2 and 3, local or regional nonprofit organizations were often important conduits for connecting RAPs to other watershed level initiatives. The realization that RAP objectives were inherently linked to the health of the surrounding watershed has been vital to advancing RAPs. For instance, early in the Rouge River RAP, stakeholders learned that to solve their problems of the release of raw sewage from 168 combined sewer overflows and urban stormwater runoff it would take collaboration among all 48 watershed communities and three counties [37]. The incorporation of the Alliance of Rouge Communities, whose purpose is to “provide an institutional mechanism to encourage watershed-wide cooperation and mutual support to meet water quality permit requirements and to restore beneficial uses of the Rouge River to the area residents”[37] exemplifies how a watershed focus can be reflected in institutional arrangements. Thus, elevating the watershed perspective is another example of how local organizations have supported and facilitated an ecosystem approach in RAPs.

### 4.2. Strategic collaborations and partnerships

Our survey highlights how many RAP institutional arrangements have grown from and evolved into a series of strategic collaborations and partnerships. Cleanup of AOCs has not been easy and required networks focused on gathering stakeholders, coordinating efforts, securing funding, and ensuring desired results are achieved. Effective collaboration in RAPs requires cooperative learning that involves stakeholders working in teams to accomplish a common goal under conditions that involve positive interdependence (i.e., all stakeholders cooperate to complete a task) and individual and group accountability [38]. In some AOCs, an integrative organization helped to foster collaboration, while in other AOCs these partnerships developed organically. For example, the Clinton River Watershed council convened stakeholders and coordinated implementation among involved stakeholders through a partnership agreement (Table 2). In contrast, the ongoing evolution of the St. Lawrence River Restoration Council at Cornwall is the product of collaboration of multiple entities and has become an integrative organization in itself (Table 2). Furthermore, collaboration, partnerships, and capacity building are critical to success of RAPs. For example, partnerships and collaborative funding were essential to achieving: over $1 billion in combined sewer overflow and urban stormwater controls in the Rouge River AOC; $180 million (Canadian) in habitat rehabilitation in the Toronto and Region AOC; $86 million of contaminated sediment remediation in the Ashtabula River AOC; and $110 million cleanup of the Severn Sound AOC [11]. Another example of innovative partnerships and collaboration among stakeholders is the four North Shore of Lake Superior AOCs (i.e., Peninsula Harbour, Jackfish Bay, Nipigon Bay, and Thunder Bay), where the Lake Superior Programs Office played an instrumental role in early RAP development and implementation.

RAPs have been the principal program to operationalize use of an ecosystem approach in management of the Great Lakes, invoking new governance paradigms [39]. Collaborative governance makes it possible to address actions that serve a public purpose by engaging groups and individuals across different institutions and spheres of society [4]. A recent analysis of connecting river RAPs found that the establishment of informal networks stemming from formal agreements resulted in individuals and groups completing partnership projects appropriate to their capacities and roles [40]. Key partnership success factors include effective working relationships, trust among partners, clarity of roles and responsibilities, well-recognized benefits to all, and effective facilitation.

#### 4.2.1. Partnering with scientific institutions has strengthened science-policy-management linkages and can cultivate local scientific capacity

The results from our survey suggest that the involvement of universities and local science centers in RAP institutional arrangements has helped to strengthen the science-policy-management connection in the AOC. Although some RAP institutional structures have scientists as members, that is not always the case. Linkages to universities and research centers are needed to help understand cause-and-effect relationships, translate science, evaluate remedial options, track progress, set priorities, and implement actions in an iterative fashion for continuous improvement [11]. We chose to distinguish partnerships with scientific institutions from other strategic partnerships because it is required to implement adaptive management and highlights the benefit of building local scientific capacity that can contribute to that management. From our survey, we found that RAP institutions that did this best sought, created, and acted on opportunities to cultivate local scientific capacity that enhanced the value of science and adaptive management outlined in the RAP. Some examples include the St. Lawrence River Institute of Environmental Science that has bolstered local research capacity at the St. Lawrence at Cornwall AOC, efforts by Lakehead University to centralize information and facilitate restoration of the North Shore AOCs on the InfoSuperior website they maintain, Canada Centre for Inland Waters’ applied research and science transfer that supports the Hamilton Harbour RAP, the Great Lakes Institute for Environmental Research of the University of Windsor that has performed necessary research in support of removing beneficial use impairments, and the River Raisin Institute’s centralization and synthesizing of water quality data into actionable plans that contribute to watershed restoration (Tables 2 & 3).

### 4.3. Promoting sustainability and fostering a stewardship ethic

Concern among some RAP practitioners is that AOC delisting can result in loss of momentum due to lack of a tangible reason to organize, loss of important sources of funding, and less frequent environmental monitoring [23]. As cleanup nears completion, many AOCs are considering “life after delisting” and exploring how to leverage remedial and preventive actions to advance broader social and economic revitalization in waterfront areas [41]. In total, our survey found that 30 of the 43 AOCs mention “life after delisting” and/or sustainability (Tables 2 and 3). This suggests the purpose of institutional arrangements are evolving beyond the original goal of delisting an AOC, into a longer-term focus on sustainability. Language regarding sustainability or life after delisting was often found in the mission statements or objectives of local organizations involved with the RAP. The tangible effect of this language has been a concerted effort to foster a stewardship-minded populace. In recent years, more emphasis is being placed on how AOC cleanup leads to reconnecting people to waterways that then leads to waterfront revitalization [42,43]. As AOC communities look to the future, local organizations will likely play a key role in reconnecting people to their restored waterfront in order to promote community revitalization.

### 4.4. Factors related to static institutional arrangements

Institutional arrangements remained relatively static in eight AOCs (i.e., Torch Lake, MI, Deer Lake, MI, Manistique River, MI, Lower Menominee River, MI/WI, Waukegan Harbor, IL, Wheatley Harbour, ONT, Oswego River, NY, and Port Hope, ON). This lack of substantial change does not equate to lack of RAP progress as three of these AOCs have been delisted (i.e., Deer Lake, Oswego River, and Wheatley Harbour) and two have completed all management actions identified in their Stage 2 RAPs (i.e., Lower Menominee River and Waukegan Harbor). In general, we believe that the lack of change indicates that the original institutional structures were well-suited to handle challenges and implement remedies. For instance, Waukegan Harbor’s institutional structure was broad and effective in building partnerships with capacity-building organizations. The problems in Waukegan Harbor were also clearly defined with regulatory agencies playing the key role in the cleanup of PCB-contaminated soils and sediment, resulting in all management actions being completed within five years of the Stage 2 RAP [44]. Cleanup remedies with clear and well recognized regulatory program responsibilities may also explain the lack of institutional evolution in some AOCs. Indeed, a majority of the eight AOCs with stable institutional structures had use impairments and problems that were being addressed under regulatory cleanup programs like the Comprehensive Environmental Response, Compensation, and Liability Act (CERCLA, i.e. Superfund) or the Resource Conservation and Recovery Act (RCRA) in addition to being an AOC. In Port Hope, Ontario, cleanup of radionuclide waste is being undertaken through regulatory programs of the Canadian federal government. Regulatory programs are inherently top-down driven processes and can rely on enforcement and liability to initiate clean ups [45]. In these AOCs, regulatory cleanup programs took precedence over voluntary efforts. Furthermore, federal funds and responsible parties typically finance regulatory cleanups so there is less need for creative and collaborative financing by local organizations.

### 4.5. Comparison with other experiences throughout the world

Comparing the nature, mechanisms, and level of public participation in RAPs to conservation management initiatives elsewhere in the world can shed light on common challenges and potentially useful strategies for overcoming barriers to effective implementation. The call for public involvement through PACs brought the public into the process at its outset. As PACs and institutional arrangements evolved, they became forums that helped establish trust amongst stakeholders, promoted shared learning and decision-making, and oriented decision-makers and managers towards an ecosystem approach [12]. Collaboration and inclusivity has been a guiding principle in the RAP planning and implementation process and should be an aspiration for all forms of polycentric resource governance. However, establishing and sustaining valuable public involvement in non-regulatory programs is not a challenge unique to the AOC program and its absence in a program can be a barrier to implementation. While seeking to identify governance bottlenecks in the implementation of the European Union’s Water Framework Directive, Zingraff-Hamed *et al.* [7] noted how practitioners thought public involvement was insufficient at every step of the implementation process, and that participatory decision-making was not common and even rejected at times. In the AOC program, a present challenge is in sustaining active stakeholder participation, which Holifield and Williams [14] suggest can be addressed through a committed focus to cultivating relationships between stakeholders and managers as well among stakeholders themselves. Our research speaks to the strength of relationship building and broad participation at an institutional level (Table 3), which has been touted as an important and transferable characteristic of effective governance in other sustainable resource management approaches, e.g. Integrated Strategies for Sustainable Urban Development in Barcelona [46] or the ‘Living Labs’ concept [47]. Overall, investment in building stakeholder networks and relationships at individual and institutional levels could be a particularly useful strategy for improving public participation in the implementation of collaborative resource governance.

The diverse and influential roles NGOs have in advancing and supporting RAPs has implications for voluntary conservation efforts internationally. RAPs lack regulatory authority and strong legislative mandates, so the success of RAPs is derived from a stewardship ethic that comes from the cultivation of an individual and collective sense of place—meaning a personal connection to a location [2]. Local nonprofits have been useful toward this end because many seek to connect communities to the watersheds they reside in through citizen science, educational campaigns, and engaging local leaders. The recognition of formally organized community groups as effective mechanisms for implementing bottom-up initiatives is also evident elsewhere in the world. In their survey of 63 community-based initiatives in Europe, Celata and Colleti [48] found that nonprofits are often the best-equipped form of community-based initiative in acquiring financial resources and support in conducive political environments. In areas of the world where government capacity and involvement in certain initiatives is minimal, NGOs have often filled the leadership void and have become increasingly involved with capacity development and local empowerment [49]. We found that nonprofits often served as the integrative organization in an AOC and had great flexibility in their roles, which mirrors what Celata and Colleti [48] report for the community-based conservation initiatives they studied. Our results paired with the international literature suggests that NGOs are an important component to the sustained success of non-regulatory conservation programs and can complement government involvement when present.

## 5. Conclusions

The Great Lakes Water Quality Agreement charge to use an ecosystem approach in RAPs challenged governments and other stakeholders to transform management. RAPs represent a shift in governance paradigm by sharing decision-making among stakeholders through local PACs, stakeholder groups, and other institutional structures. Experience has shown that there is no single best approach to implementing an ecosystem approach in RAP development and implementation, rather it is fair to say that there were 43 locally designed ecosystem approaches that helped involve stakeholders in a meaningful way, foster cooperative learning, share decision-making, and ensure local ownership.

Institutional arrangements in 35 of the 43 AOCs evolved since 1985 to improve effectiveness, transition to implementation, and ensure relevancy and sustainability. Since 1985, 32 of the 43 AOCs have established a nonprofit organization or worked with one that has built capacity and helped secure necessary funding. Continued research and cooperative learning are needed to help these institutional structures grow and evolve to meet long-term goals of sustainability.

Considerable work is underway throughout the world to foster use of an ecosystem approach and practice ecosystem-based management practices. It is recommended that an international conference be convened to share practical experiences in the use of an ecosystem approach in management, including its origin, status, and how it can fully maximize its impact on the Great Lakes and elsewhere.

## Figures and Tables

**Table 1.** Key characteristics of effective institutional arrangements designed to ensure stakeholder involvement and foster use of an ecosystem approach in RAP development and implementation for Great Lakes Areas of Concern (derived from literature [12,20]).

Key Characteristic	Purpose or Function
Fosters integration through a broad-based institutional structure	Engage stakeholders who impact or are impacted by pollution in the AOC, ensure meaningful involvement for use restoration, and foster integration across disciplines and among water, land, air, municipalities, industries, etc.
Promotes a watershed focus	Ensure planning and management that account for watershed boundaries
Encourages local ownership of RAP process	Structure the RAP process to create a sense of ownership by participants, who were the very businesses, state and local agencies, and citizens who had to help implement remedial and preventive actions; engage local leaders; recruit champions
Establish compelling vision and clear goals	Establish a vision that can be carried in the hearts and minds of all stakeholders and measurable targets to focus actions
Builds collaboration, partnerships, and capacity	Build institutional capacity and establish or leverage partnerships for cleanup and restoration, and foster collaborative problem-solving and financing
Connects to scientific organizations to understand problems and causes, and support scientifically-sound decision-making	Strengthen science-policy-management linkages and practice adaptive management
Celebrates a record of success for the AOC, including benefits	Showcase restoration progress publicly to build momentum and maintain community interest and buy-in for revitalization of their ecosystem
Emphasizes sustainability planning for life after delisting as an AOC	Enact remedial and preventive actions to advance broader social and economic revitalization in waterfront areas and foster a stewardship ethic

**Table 2.** Evolution of RAP institutional structures for use of an ecosystem approach in restoring Great Lakes AOCs, 1985–2019. Table entries are listed in a geographic order from east to west with exception of binational AOCs, which are listed at the end.

Area of Concern	Evolution of AOC/RAP Institutional Structures
Peninsula Harbour (Ontario)	The Public Advisory Committee (PAC) was established in 1989 to facilitate public input in RAP development and implementation. The Lake Superior Programs Office was formed in 1991 by Environment Canada, the Canada Department of Fisheries of Oceans, the Ministry of Environment and Energy and the Ministry of Natural Resources as a unique, one-window approach to deliver projects recommended by PACs for the four northshore AOCs (i.e., Thunder Bay, Nipigon Bay, Jackfish Bay, and Peninsula Harbour). The RAP is now facilitated by Lakehead University with supervision from several provincial and federal agencies. The PAC disbanded after they completed their original objectives of evaluating BUIs and developing water use goals for the AOC that could be used as community-based guidelines for the RAP. However, public involvement in the AOC continued through the development and implementation of the contaminated sediment management plan. In 2008, the Peninsula Harbour Community Liaison Committee (CLC) was formed to facilitate public involvement in the sediment management plan and other RAP discussions. The CLC, which is composed of several former PAC members, provides input to the RAP and assists with information sharing within the community of Marathon and the Ojibways of the Pic River First Nation. InfoSuperior was created in the 2000s as a research and information network for Lake Superior, including RAPs, focused on community engagement, stakeholder communication, and assisting with restoration projects. EcoSuperior Environmental Programs was established in 1985 as a nonprofit organization for environmental stewardship in Northwestern Ontario and the Lake Superior Basin through engagement, education, collaboration, action, and leadership.
Jackfish Bay (Ontario)	The PAC was established in 1989 to facilitate public input in RAP development and implementation. The Lake Superior Programs Office was formed in 1991 by Environment Canada, the Canada Department of Fisheries of Oceans, the Ministry of Environment and Energy and the Ministry of Natural Resources as a unique, one-window approach to deliver projects recommended by PACs for Thunder Bay, Nipigon Bay, Jackfish Bay, and Peninsula Harbour AOCs. The RAP is now facilitated by Lakehead University with supervision from several provincial and federal agencies. In 2008, the Public Area in Recovery Review Committee (PARRC) was established to continue the role of the original PAC as the AOC transitioned to Area in of Concern in Recovery (AiR) status. The PARRC has helped outline a plan for guiding the AiR through recovery to delisting and has been critical to defining what AiR status means. Among other recommendations, the PARRC has highlighted the importance of ensuring ongoing community engagement and education during the AiR phase. InfoSuperior serves as an important mechanism for facilitation and information sharing. EcoSuperior Environmental Programs is a nonprofit organization established in 1985 for regional environmental stewardship through engagement, education, collaboration, action, and leadership.
Nipigon Bay (Ontario)	The PAC was established in 1989 to facilitate public input in RAP development and implementation. The Lake Superior Programs Office was formed in 1991 by Environment Canada, the Canada Department of Fisheries of Oceans, the Ministry of Environment and Energy and the Ministry of Natural Resources as a unique, one-window approach to deliver projects recommended by PACs for Thunder Bay, Nipigon Bay, Jackfish Bay, and Peninsula Harbour AOCs. InfoSuperior serves as an important mechanism for facilitation and information sharing. EcoSuperior Environmental Programs is a nonprofit organization established in 1985 for regional environmental stewardship through engagement, education, collaboration, action, and leadership.
Thunder Bay (Ontario)	The PAC was established in 1989 to facilitate public input in RAP development and implementation. The Lake Superior Programs Office was formed in 1991 by Environment Canada, the Canada Department of Fisheries of Oceans, the Ministry of Environment and Energy and the Ministry of Natural Resources as a unique, one-window approach to deliver projects recommended by PACs for Thunder Bay, Nipigon Bay, Jackfish Bay, and Peninsula Harbour AOCs. The RAP is now facilitated by Lakehead University with supervision from several provincial and federal agencies. InfoSuperior serves as an important mechanism for facilitation and information sharing. EcoSuperior Environmental Programs is a nonprofit organization established in 1985 for regional environmental stewardship through engagement, education, collaboration, action, and leadership.
St. Louis River (Minnesota and Wisconsin)	A Citizen Advisory Committee (CAC) was established in 1985 to ensure public input for the RAP and to help foster use of an ecosystem approach. In 1996, the CAC was incorporated as a nonprofit organization called the St. Louis River Alliance (SLRA). SLRA works to oversee activities and practices that are helping to restore, protect, and enhance the St. Louis River.
Torch Lake (Michigan)	A Public Action Council was established in 1997 and within one year became incorporated as a nonprofit organization. The Council advised on restoration and has successfully raised funds from local sources to defray logistical costs. Restoration benefited from the Superfund Program, including U.S. Environmental Protection Agency (EPA), U.S. Department of Agriculture’s Natural Resources Conservation Service (NRCS), and Michigan Departments of Environmental Quality (DEQ) and Natural Resources. U.S. EPA entered into an interagency agreement with NRCS that allowed it to leverage the NRCS’s soil erosion/bank stabilization expertise, site familiarity, and existing rapport with the community.
Deer Lake and Carp River and Creek (Michigan)	A PAC was established in 1987 to ensure public input on RAP development and implementation. The Council worked with the State of Michigan to achieve delisting as an AOC. The City of Ispeming has committed to long-term stewardship of Partridge Creek because it owns the property.
Manistique River and Harbor (Michigan)	Public meetings were held in 1986 and 1987. In 1993, a Public Advisory Council was established, and U.S. EPA started engaging potentially responsible parties (Manistique Papers and Edison Sault Electric Company) for remediation of PCB-contaminated sediment. Early remediation of contaminated sediments was led by the Superfund program. The RAP process was primarily a government-led process, with the federal government leading remediation of contaminated sediment, the Michigan Department of Environmental Quality (MDEQ) serving as the RAP coordinator, and City of Manistique’s wastewater superintendent serving as the Council chair. The Council adopted delisting guidelines in 2005 and a committee was established to address habitat under the Council. The City of Manistique has taken the lead on championing the Manistique River as a tourist destination and built a boardwalk to reconnect people to this waterway.
Lower Menominee River (Wisconsin and Michigan)	The CAC was formed in 1988 as a means of incorporating stakeholder feedback into the RAP and to serve as ambassadors on AOC issues for the Marinette and Menominee communities. The CAC developed governing bylaws in June of 2011 to ensure the committee’s long-term viability and balanced community representation. CAC members also played a critical role in community engagement and outreach. Representatives from the Wisconsin Department of Natural Resources (DNR) and Michigan MDEQ, and each municipality, serve on the technical advisory committee (TAC), along with representatives from academia, the private sector, and utilities.
Fox River and Southern Green Bay (Wisconsin)	Wisconsin DNR established a CAC in 1986 for public involvement in the RAP and to ensure use of an ecosystem approach. Four TACs supported RAP development. The original CAC was disbanded in 1988 and replaced with an Implementation Committee. University of Wisconsin-Extension helped facilitate education and outreach. The Green Bay community’s strong sense of place is reflected in the institutional arrangement surrounding the RAP. In reference to the community’s football team, the CAC adopted the name “The Clean Bay Backers” as a means of evoking loyalty to their water resources much like the community’s loyalty to their football team. The CAC has been active in community engagement, including an annual “Bringing Back the Bay Tour” in which they take local officials and community leaders on an area tour and inform them on ecosystem problems. The CAC includes individuals from the general public, municipalities, academia, and nonprofit organizations, including Ducks Unlimited and the FoxWolf Watershed Alliance.
Sheboygan River (Wisconsin)	In 1984, the Sheboygan County Water Quality Task Force was created by citizens concerned about pollution in the AOC. This Task Force was used to ensure public input in RAP development, with support from the Sheboygan River and Harbor TAC and a Wisconsin DNR Workgroup for river cleanup. The Task Force eventually evolved into a CAC. The University of Wisconsin Extension helped with public education and outreach and helped facilitate the CAC and TAC. Sheboygan River Basin Partnership (SRBP) was created in 1998 as a nonprofit alliance of conservation/environmental groups in the watershed. SRBP’s mission is to cultivate partnerships to raise public awareness, increase participation in stewardship, and promote sound environmental decision making for the river. Water Action Volunteers, a collaborative citizen program under the Wisconsin DNR and University of Wisconsin Extension, performed citizen science to assess ecosystem health. Waterfront revitalization is now being championed by the City of Sheboygan and the Sheboygan County Economic Development Corporation.
Milwaukee River Estuary (Wisconsin)	Starting in 1987, Wisconsin DNR and a TAC began developing the RAP, with input from a CAC and Citizen Education and Participation Subcommittee. The CAC provided input on RAP goals, established a long-term vision, advised on RAP implementation, and served as a unifying entity for all AOC stakeholders. By the mid-1990s the TAC and the CAC stopped meeting, but RAP development continued through a steering committee. However, the RAP lacked a true stakeholder coalition. In 2012, Wisconsin DNR and University of Wisconsin-Extension established a new CAC with broader participation. University of Wisconsin-Extension facilitated this process with grant support from U.S. EPA. Today, the CAC is comprised of public, private, and nonprofit representatives to provide two-way communication between AOC staff and member organizations. Milwaukee Riverkeeper is a nonprofit organization focused on monitoring water quality and providing science-based advocacy for the Milwaukee River. Water Action Volunteers, a collaborative citizen program of Wisconsin DNR and University of Wisconsin Extension, performs citizen science to assess ecosystem health.
Waukegan Harbor (Illinois)	In 1990, a Citizens’ Advisory Group (CAG) was established to assist Illinois Environmental Protection Agency in RAP development and implementation. It was structured to include corporate, governmental, shipping, environmental, and public representatives. The CAG’s institutional structure was broad and diverse early on with 26 member-organizations in 1990 and was increased to 34 member-organizations in 2019. The CAG has been instrumental in obtaining cooperation from local parties regarding investigations, gaining access from private businesses, and securing federal grant money. The CAG also promotes redevelopment and stewardship of the lakefront and works with others to protect Waukegan Harbor as a local and regional asset.
Grand Calumet River (Indiana)	Citizens Advisory for the Remediation of the Environment (CARE) was established in 1987 to advise Indiana Department of Environmental Management on restoration of uses in the AOC. The committee is comprised of members of local organizations, industries, academia, and agencies dedicated to improving the chemical, physical, and biological integrity of the area’s ecosystem. CARE also educates the public about AOC progress. The Grand Calumet River Restoration Fund (GCRRF) was established through a settlement with industrial users in the Sanitary District of Hammond. Its purpose is to address and correct environmental contamination in the AOC, including the cleanup of contaminated sediment and the remediation of natural resource damages. GCRRF was established by a memorandum of understanding between state and federal partners and is authorized to conduct studies and make decisions for the management and administration of funds. Chicago Wilderness Alliance is sponsored by the Friends of the Forest Preserves, which is a nonprofit organization formed in 1996. It has more than 260 public and private partners involved in restoring, protecting, and connecting 728,400 ha, including portions of the Grand Calumet River through conservation and sustainable development practices.
Kalamazoo River (Michigan)	In 1987, the Michigan DNR developed the RAP with funding from the State of Michigan. The City of Kalamazoo formed a coalition of stakeholders and agency representatives known as the Kalamazoo River Basin Strategy Committee (Basin Committee). The Basin Committee helped implement and coordinate the RAP and a fisheries management plan. In 1993, the Kalamazoo River Public Advisory Council was established to fill this institutional niche. In 1998, the Council’s function was assumed by the nonprofit organization Kalamazoo River Watershed Council (KRWC) that has established broader goals that go beyond delisting. In 2001, the Kalamazoo River/Lake Allegan Watershed Phosphorus Reduction Committee was established to complement the PAC and oversee coordinated efforts to establish Total Maximum Daily Loads to comprehensively address nonpoint sources.
Muskegon Lake (Michigan)	In 1985, a Public Advisory Council was established to ensure public participation in the RAP and help implement an ecosystem approach. In the early 1990s the Muskegon Lake Watershed Partnership was established as a community-based, volunteer partnership organization to support grassroots, local, state, regional, federal, and international programs to restore Muskegon Lake and the Great Lakes. This Partnership has developed a Muskegon Lake Ecosystem Action Plan to facilitate the continuation of coordinated, natural resources stewardship of Muskegon Lake and Lower Muskegon River Watershed from 2018 through 2025.
White Lake (Michigan)	The RAP was developed by state and federal agencies, local government officials, stakeholder groups, and independent citizens. In 1992, the Muskegon office of the Lake Michigan Federation (now the Alliance for the Great Lakes) obtained a grant that officially established a Public Advisory Council. The Lake Michigan Federation provided administrative support to the Council in the early years, but later transferred that role to the Muskegon Conservation District (MCD) who served as a fiduciary and provided technical support to the PAC through delisting. U.S. EPA provided funds to support a position for three years at the MCD to set up an environmental stewardship program. This program focuses on community partnerships for long-term conservation, preservation, and restoration of White Lake. MCD has helped foster use of an ecosystem approach and achieve local ownership. White Lake was delisted as an AOC in 2014, but the PAC has remained active and has developed a strategic plan for 20152018 that established the White Lake Environmental Network – a network of stakeholders focused on sustaining momentum for revitalization.
Saginaw River and Bay (Michigan)	In 1987, the Saginaw Basin Natural Resources Steering Committee was established to provide public input on the RAP. Today, the board of directors of the Partnership for the Saginaw Bay Watershed (Partnership) serves as a Public Advisory Council. The Partnership is a nonprofit organization that is comprised of public and nongovernmental stakeholders across the Saginaw River Basin. This Partnership merged the Saginaw Basin Alliance and the Saginaw Bay Watershed Council in 1995, and facilitates intergovernmental coordination and public involvement, provides guidance on public policy, and assists with restoration projects in the basin. The Partnership is structured and functions as a collaborative for watershed management. The Saginaw Bay Watershed Initiative Network (WIN) is a community-based, voluntary initiative that connects people, resources, organizations, and programs. Implementation of the RAP has advanced with funding acquired through WIN with financial support from 11 foundations.
Collingwood Harbour (Ontario)	The Public Advisory Committee (PAC) was established in 1987 to ensure public input in RAP development and foster use of an ecosystem approach. The PAC was incorporated in 1993 with an office located in downtown Collingwood. A storefront called The Environment Network of Collingwood opened for the RAP to serve as a central location for RAP activities. Several years later, the name was changed to The Environment Network. The Network went on to develop a strategic plan called the Greening of Collingwood that championed pollution prevention for residents, businesses, and industries. To this day the Network operates as a cooperative, providing people with opportunities for work and a place for people to learn how they can operate their business or home in an ecologically, socially, and economically sustainable manner.
Severn Sound (Ontario)	The RAP was initiated in the mid-1980s through a partnership agreement between the federal and provincial governments and municipalities in the Severn Sound area. The partnership became the Severn Sound Environmental Association (SSEA) – a Joint Municipal Services Board Ontario Municipal Act, Section 202, representing the 10 municipalities in the Severn Sound area. SSEA played a key role in sewage treatment plant upgrades, farm pollution control projects, stormwater treatment studies, tree planting, shoreline restoration and ecosystem monitoring, and public outreach on environmental issues. SSEA helped provide community-based and cost-effective environmental management for the AOC, which helped sustain momentum and achieve delisting. Following delisting, creative local partnership agreements and financing were arranged to continue long-term implementation and to meet emerging environmental and sustainability challenges.
Spanish River (Ontario)	A PAC was established in the late 1980s to assist with RAP development and promote use of an ecosystem approach. Early on the effectiveness of the PAC was hampered by poor working relationships resulting from disputes and disagreements among stakeholders [22]. These issues were addressed, and a coordinated approach was achieved among the federal and provincial governments and the PAC that led to implementation of all recommended actions and being designated as an “AOC in recovery” in 1999. In 1994, a group of concerned citizens formed the nonprofit organization called the Friends of the Spanish River (FOSR). FOSR was active in the RAP and worked closely with all stakeholders on community engagement, RAP implementation, and public education. FOSR disbanded in 2013, having achieved their mandate.
Clinton River (Michigan)	A Public Advisory Council was established in 1986 and worked closely with stakeholders to ensure use of an ecosystem approach in the RAP process. The Council served as an integrative organization among stakeholders, delineated specific remedial actions to be implemented by various entities through a “partnership agreement”, and tracked progress. The Clinton River Watershed Council (CRWC), a nonprofit organization established in 1972, has provided grassroots coordination from the outset of the RAP process. CRWC has coordinated local restoration efforts and provided administrative and technical support to the Council. CRWC has also helped foster a “sense of place” and local ownership through engaging and educating the public on watershed issues and leveraging the river’s “placemaking” potential to support community development.
Rouge River (Michigan)	In 1985, the Rouge River Basin Committee was established to develop and implement the RAP with representation for all 48 watershed communities. In 1986, the Friends of the Rouge was established to raise awareness and promote cleanup of the Rouge River. In 1992, the representatives of the Basin Committee were reorganized into the Rouge RAP Advisory Council and in 1993 the Rouge River National Wet Weather Demonstration Project was established with over $350 million to help implement CSO controls and innovative storm water management techniques called for in the RAP. In 2003, the Alliance of Rouge Communities was founded to help implement comprehensive monitoring, facilitate communication among watershed stakeholders, and coordinate sub-watershed planning to implement stormwater plans.
River Raisin (Michigan)	Since 1985, Michigan DNR and DEQ have worked with a Public Advisory Council to ensure public involvement and local ownership of the RAP, and have coordinated with the River Raisin Watershed Council, the City of Monroe, and many others. In 2006, the city established the Commission on the Environment and Water Quality and nested the River Raisin Public Advisory Council under this commission, ensuring a long-term commitment to both restoration and life after delisting within the city’s governmental structure. The River Raisin Institute was established by the Immaculate Heart of Mary (IHM) Sisters to revitalize and preserve sustainable ecosystems, including the River Raisin AOC. The Institute, the River Raisin Watershed Council, and Public Advisory Council collaborate on watershed management and sustainability.
Maumee River (Ohio)	The first public meeting for the RAP was convened by Ohio Environmental Protection Agency in 1987. Out of this initial meeting came a RAP Advisory Committee made up of a broad cross section of business, government, community, and nongovernmental stakeholders. Key assistance was provided by Toledo Metropolitan Area Council of Governments. Later the name was changed to the Maumee River AOC Advisory Committee. A critical partner in the success of the Maumee RAP has been the Duck and Otter Creeks Partnership formed in 1999. The Partnership worked very closely with the Maumee RAP to develop the Stage II RAP for the Maumee AOC.
Black River (Ohio)	In the early 1990s, a committee called the Black River RAP was established as a unique community-based public/private initiative for restoration of impaired uses. Its motto was “Our River, Our Responsibility.” Based on guidance from the U.S. EPA, the name was changed to the Black River AOC Advisory Committee in 2014. The Black River Facilitating Organization (BRFO) identifies and focuses on priorities within the Black River AOC, supports agencies involved in restoration and remediation, facilitates public outreach, and promotes watershed education. The BRFO raises funds, manages programs and projects, and coordinates and assists the Advisory Committee. During early RAP development, Seventh Generation was established as a nonprofit organization to promote environmental education and help people see how their decisions would impact the next seven generations, consistent with Iroquois Nation beliefs. One of their accomplishments before closing was the creation of the Black River Environmental Center.
Cuyahoga River (Ohio)	In 1988, the Ohio Environmental Protection Agency (EPA) appointed a 33-member planning committee to develop the Cuyahoga River RAP. This organization, called the Cuyahoga River RAP Coordinating Committee was made up of a balanced representation of stakeholders in the planning and implementation process. In 1989, the nonprofit Cuyahoga River Community Planning Organization (later renamed Cuyahoga River Restoration) was created to support the RAP’s activities. Today, Cuyahoga River Restoration continues to support efforts to restore, revitalize, and protect the Cuyahoga River watershed and nearshore are of Lake Erie. In 2012, Flats Forward was created as nonprofit organization for community and economic development.
Ashtabula River (Ohio)	In 1988, a 30-member RAP Advisory Council was established by Ohio Environmental Protection Agency to ensure public participation in the RAP using an ecosystem approach. In 1994, a group of federal, state, local, and private entities came together to form the Ashtabula River Partnership (ARP) to foster cooperative approach to remediation of contaminated sediments within the AOC. The ARP was an important institutional mechanism that played a critical role in project success. This partnership pooled technical resources, worked together to build a consensus approach to remediation of contaminated sediment that plagued the AOC for the better part of four decades, and utilized a mix of public and private funding sources to address the problem. The ARP also served as a forum for coordination among the key players, meaningful engagement of the impacted community in problem resolution, and streamlined technical discussions and permitting processes.
Presque Isle Bay (Pennsylvania)	A Public Advisory Committee and Pennsylvania Department of Environmental Protection (DEP) were the driving forces behind the RAP process. While the Committee officially formed in 1991, the foundation of stakeholder involvement had been present before AOC designation. The concerned citizens that advocated for Presque Isle Bay to be designated an AOC in 1991 joined this Committee. Joining the Committee was an established delegation created by the Mayor of Erie and Erie County Executive called the Erie Harbor Improvement Council. A second evolution of the Committee occurred following the delisting of the AOC in 2013. The Committee then joined the Pennsylvania Great Lakes Lake Erie Environmental Forum (Forum), which was formed by the Pennsylvania DEP with the help of Pennsylvania Sea Grant to engage and inform the public. The Forum then expanded its focus to include the entire watershed. Supporting the Forum and environmental stewardship are Pennsylvania Sea Grant, Pennsylvania DEP, Environment Erie, Erie County Conservation District, Pennsylvania Lake Erie Watershed Association, and the Regional Science Consortium at Presque Isle [23]. Waterfront revitalization is now being championed by the City of Erie, the Port Authority, and Erie County.
Wheatley Harbour (Ontario)	Lacking a formal PAC, the Wheatley Harbour RAP was developed through a multi-institutional partnership, including Environment and Climate Change Canada, several provincial agencies, the Essex Region Conservation Authority, and the Essex County Stewardship Network. The community was engaged on an “as needed” basis throughout the RAP process. After delisting in 2010, monitoring and maintenance activities have been conducted under the auspices of on-going, governmental programs, such as provincial fish contaminant monitoring, Lake Erie’s Lakewide Action and Management Plan, and federal programs [23].
Buffalo River (New York)	From 1985 through the early 2000s, the New York State Department of Environmental Conservation (DEC) served as the RAP coordinator, with public participation and input from a Remedial Advisory Committee. In 2003, the Buffalo Niagara Waterkeeper was the first nonprofit organization in the Great Lakes selected to re-energize the RAP process, coordinate implementation, and catalyze further progress, ensuring life after delisting. Since 2012, this unique partnership has raised $56.5 million (U.S.) for contaminated sediment remediation and $25 million (U.S.) for habitat rehabilitation.
Eighteen Mile Creek (New York)	The RAP was developed by New York State DEC, in cooperation with the Remedial Advisory Committee, which was comprised of local officials, landowners, and stakeholders selected by the New York State DEC commissioner. In 2005, the Niagara County Soil & Water Conservation District (NCSWCD) took over as the lead agency for the RAP. The NCSWCD helped coordinate remedial actions among federal, state, and local partners and provides staff support to the Committee that provided input on RAP implementation and ensured public outreach.
Rochester Embayment (New York)	A comprehensive water quality management structure was in place in Monroe County prior to the onset of the RAP process and provided an existing institutional framework for the RAP. The RAP was written by and coordinated by the Water Quality Management Agency (WQMA) of the Monroe County Health Department. The Monroe County Water Quality Coordinating Committee (WQCC) and analogous committees in other counties provided technical expertise to the WQMA and coordinated task groups. The Water Quality Management Advisory Committee (WQMAC), in existence since 1979, was the primary mechanism for public input on the RAP. Regional organizations helped connect surrounding counties to the RAP process, while also connecting the RAP to larger watershed programs. Primary among these were the Water Resources Board (WRB), which is the governing body of the Finger Lakes – Lake Ontario Watershed Protection Alliance (FL-LOWPA). FL-LOWPA is an alliance of 24 New York counties in the Lake Ontario Basin and provides additional resources to the RAP and helps coordinate the RAP with other watershed, management programs that address local water priorities. The RAP formed the Water Education Collaborative (WEC) as a nonprofit organization and the Stormwater Coalition of Monroe County. WEC administers public education programs that support the RAP and water quality initiatives in the community. The Stormwater Coalition is an intermunicipal agreement with 29 institutions that work collaboratively to comply with Federal stormwater regulations to improve water quality. WEC and the Stormwater Coalition provide a foundation for stewardship and revitalization following future delisting as an AOC.
Oswego River (New York)	The New York State DEC formed a Citizen’s Advisory Committee in 1987 which was tasked it with providing public input on the RAP. The Committee was comprised of local residents, industrial representatives, outdoor enthusiasts, scientists, environmentalists, and local governmental representatives. In 1991, the Committee was replaced by a Remedial Advisory Committee (RAC), which had a similar composition as the Committee, but also aided the New York State DEC in RAP implementation. The RAC dissolved following delisting in 2006. Post-delisting momentum has been maintained by city and county governments [23], and the focus of environmental stewardship has expanded beyond the original AOC boundaries to focus on the larger watershed. This watershed focus is consistent with the earlier RAP’s approach that considered upstream remedial actions that affected use impairments within the AOC.
Bay of Quinte (Ontario)	In 1987, a PAC was established to facilitate public input on RAP development. Since 1997, implementation of recommended actions for the Bay of Quinte area has been facilitated by members of the Bay of Quinte Restoration Council, including representatives from the Lower Trent Region Conservation, Quinte Conservation, Environment and Climate Change Canada, Ontario Ministry of the Environment, Conservation and Parks, Ontario Ministry of Natural Resources and Forestry, Fisheries and Oceans Canada, Ontario Ministry of Agriculture and Rural Affairs, Mohawks of the Bay of Quinte, local communities, and Canadian Forces Base Canada.
Port Hope (Ontario)	Port Hope is a unique situation because of legacy contamination with low-level radioactive waste materials from historical mining and refining operations between 1933 and 1953. Cleanup has been led by the Government of Canada and the Port Hope Area Initiative. Throughout the environmental assessment phase, there was extensive public consultation, and public engagement continues through a Citizen Liaison Group.
Toronto and Region (Ontario)	In 1987, Environment Canada and Ontario Ministry of the Environment established a PAC to facilitate public input in RAP development. Today, the Toronto and Region RAP is managed by representatives from Environment and Climate Change Canada (ECCC), the Ontario Ministry of the Environment, Conservation, and Parks (MECP), the Ontario Ministry of Natural Resources and Forestry (MNRF), Toronto Water, and the Toronto and Region Conservation Authority (TRCA). Since 2002, TRCA has led the administration of the RAP under an agreement with ECCC and the MECP. The RAP team works with many partners, including nonprofits organizations like Waterfront Toronto.
Hamilton Harbour (Ontario)	In 1985, a Hamilton Harbour stakeholder group was established to ensure public participation in the RAP and help implement an ecosystem approach. A scientific Writing Team made up of federal and provincial scientists and resource managers prepared RAP reports. The Writing Team reported their findings to the Stakeholder Group for input. No distinction was made between decision-making authority of the Stakeholder Group or the Writing Team; they worked by consensus. Upon completion of the Stage 2 RAP in 1992, the Stakeholder Group disbanded and defined two groups to take its place; the Bay Area Implementation Team (BAIT) and the Bay Area Restoration Council (BARC). BAIT includes all the agencies, institutions, and corporations who accepted the responsibility for implementing recommended remedial actions. BARC is an independent incorporated citizens group responsible for monitoring progress on the remedial actions and to educating and advocating for remedial actions. Neither BAIT nor BARC were identified as subordinate to the other. BAIT and BARC continue to work to clean up and restore Hamilton Harbour as a thriving, healthy, and accessible ecosystem.
St. Marys River (Michigan and Ontario)	A binational PAC (or BPAC) was established in 1988 with various stakeholder groups from the United States and Canada. The BPAC has been active in fostering partnerships with other community groups that focus on the restoration and protection of the St. Marys River ecosystem. Through these relationships, the BPAC identifies and prioritizes programs and projects that can help achieve the goals of RAP. A priority list of these projects has been incorporated into the Lake Superior Lakewide Action and Management Plan (LAMP). The BPAC operates out of an office at Lake Superior State University. The BPAC’s partnership with the University has increased the BPAC’s capacity by involving students in RAP-related activities such as maintaining the BPAC’s website and interactive map explorer for visualizing RAP progress. The BPAC also supported the creation of the nonprofit organization: Friends of the St. Marys River (FOSM). In addition to supporting the RAP, FOSM works to promote and protect the river’s value as a Canadian Heritage River, which was granted to it in 2000. Through the establishment of FOSM and the network of stewardship-focused stakeholder groups, the BPAC is fostering a culture of local involvement in environmental planning that should persist beyond delisting. In 2008, the St. Mary’s Water Quality Network was formed to promote public outreach efforts regarding the fish tumors impairment.
St. Clair River (Michigan and Ontario)	In 1988, a BPAC was established by U.S. EPA, Environment Canada, Michigan DNR, and Ontario Ministry of the Environment to advise and oversee a RAP team during the planning, adoption, and implementation. The BPAC serves as an interface between the public and the RAP team. RAP team members and BPAC members compose four task teams. Also, in 1988 two nonprofit organizations were formed under the name of Friends of the St. Clair River (FOSCR) to support BPAC efforts in Canada and the United States. FOSCR started as a fiduciary for BPAC, but over time evolved to foster public education and community engagement. Together, FOSCR and BPAC have made public education and community engagement a priority from the outset. In 2005, the Canadian Remedial Action Plan Implementation Committee was established to guide implementation of the remaining remedial actions on the Canadian side of the AOC. U.S. EPA and Michigan DEQ informally participate in this committee as needed.
Detroit River (Michigan)	A BPAC was established in 1987 at the onset of Stage 1 RAP development. It soon was paralyzed by lack of trust and ineffective governance and split apart into separate U.S. and Canadian public involvement processes. In 1991, the Detroit River Public Advisory Council (PAC) was established in the United States to facilitate public involvement in cleanup efforts. Also, in the early 1990s the Friends of the Detroit River were established as a nonprofit organization to enhance public involvement in the RAP. Today, the PAC continues to facilitate public participation in the RAP and assists with its implementation, under the direction of the Friends of the Detroit River. U.S. PAC members now periodically participate in Detroit River meetings in Canada.
Detroit River (Ontario)	A BPAC was established in 1987 for RAP development. With the dissolution of BPAC, the Detroit River Canadian Cleanup (DRCC) was established in Ontario in 1998 with federal and provincial funding. A RAP Coordinator works out of the Essex Region Conservation Authority office, provides support for RAP participants, and liaises with U.S. RAP participants on an ongoing basis. DRCC includes a local, provincial, and federal agency personnel who have RAP implementation responsibility, as well as private sector, academic, and nongovernmental partners. DRCC also has an active PAC that includes participants from Essex County Field Naturalists’ Club, Little River Enhancement Group, Citizens Environment Alliance, Unifor (formerly Canadian Auto Workers) and the Windsor and District Labour Council, among others. DRCC and PAC members periodically participate in Detroit River meetings in the U.S.
Niagara River (Ontario)	In 1988, a PAC was established by Environment Canada and Ontario Ministry of the Environment to ensure stakeholder participation in the RAP process and to foster use of an ecosystem approach. In 1998, it was incorporated as a nonprofit organization called the Niagara River Restoration Council (NRC). The NRC is based out of Welland, Ontario, but is active in the implementation on both sides of the river. The NRC has carried out habitat rehabilitation projects, including the construction of stream buffers and the removal of barriers to fish migration throughout the Niagara region. In 1999, the Niagara Peninsula Conservation Authority (NPCA) took an active leadership role and became the host organization for administering and coordinating the RAP with funding support from the federal and provincial government and continues today to fulfill these secretariat services. The NPCA has been an active participant in the RAP initiative since its inception in the late 1980s and has completed many remedial and preventive actions. Although there are separate RAPs for the Ontario side and the New York side, they are formally connected through an international advisory committee [24]. In addition, the Niagara River Toxics Management Plan is binational in scope and has been instrumental in reducing toxic substances’ loadings.
Niagara River (New York)	New York State DEC has served as the RAP coordinator since 1985. The expansion of the former Friends of the Buffalo River (established in 1989) into the Buffalo Niagara Waterkeeper helped to integrate the Niagara River RAP into regional efforts to clean up the larger watershed, which includes several other AOCs. In 2004, the Niagara River Greenway Commission was established to preserve and enhance the Niagara River Greenway, while emphasizing economic development activities. Although there are separate RAPs for the Ontario side and the New York side, they are formally connected through an international advisory committee (Niagara River RAP Stage 2, 1995). In addition, the Niagara River Toxics Management Plan is binational in scope and has been instrumental in reducing toxic substances’ loadings.
St. Lawrence River at Massena and Akwesasne (New York)	New York State DEC is the lead agency for the St. Lawrence River at Massena RAP. Government agencies and potentially responsible parties identified in the Superfund process were most involved in the initial stages of the RAP. DEC formed a Citizen Advisory Committee in the late 1980s to advise on RAP development. The Committee’s focus on evaluating the RAP process in the early stages produced more efficient operating procedures and effective conflict management mechanisms. The CAC disbanded after the submission of the Stage 2 RAP and was replaced by a Remedial Advisory Committee with a charge to provide advice on RAP implementation and restoration targets and ensuring that stakeholders’ interests and concerns addressed. The AOC was then renamed the St. Lawrence River at Massena and Akwesasne to reflect the involvement of the Akwesasne Mohawk Nation.
St. Lawrence River at Cornwall (Ontario)	The RAP team was formed in 1986, consisting of agency representatives from Environment Canada and Ontario Ministries of the Environment and Natural Resources. In 1988, a PAC was formed for public involvement in the RAP. The PAC’s desire for a locally based research institute encouraged the creation of the St. Lawrence River Institute of Environmental Science (SLRIES) in 1992. SLRIES has bolstered community engagement and local research capacity in part due to the partnerships they have fostered with regional universities. In 1997, the PAC disbanded and was replaced with the Cornwall and District Environment Committee (CDEC), which currently serves as a public watchdog overseeing the implementation of the RAP, while also monitoring other environmental issues [25]. The St. Lawrence River Restoration Council (SLRRC) was originally formed in 1989 as forum for information sharing among the involved parties, which included members from the Cornwall PAC, several of their U.S. counterparts from Massena Citizen Advisory Committee, and the Mohawks Agree on Safe Health (MASH) group. The original SLRRC disbanded in 1991. In 1998 it was reinstated with a new purpose focused on oversight and accountability in the implementation of the 64 recommended Canadian remedial actions. The new SLRRC integrated governments and local stakeholders into a single institution – contrasting the earlier approach of separate entities (i.e., RAP team, PAC, and former SLRRC). The SLRRC is currently led by the Raisin Region Conservation Authority. As cleanup and the relative priority of environmental issues has changed over the last decade, the SLRRC has sought to re-examine its mandate, responsibilities, and scope of activity. In 2014, SLRRC evolved into a new organization serving as a river-related environmental communication, education, and coordination hub for future activities on the St. Lawrence River in eastern Ontario. The SLRRC aims to organize a comprehensive network that supports ongoing research and remediation on the river, while at the same time encouraging an environmentally balanced and sustainable approach to recreation and development.

**Table 3.** Main findings of common institutional changes in Areas of Concern (AOCs) that promote the use of an ecosystem approach as deduced from survey results. Each finding is contextualized by the corresponding relevant key characteristic(s) presented in Table 1 and select examples.

Common changes in institutional arrangements that advanced the use of an ecosystem approach and other conclusions	Relevant Key Characteristic(s)	Examples
Increased prevalence of local nonprofits acting as an integrative organization for the institutional arrangement	Encourages local ownership of RAP process	• Muskegon Lake: Muskegon Lake Watershed Partnership • Severn Sound: Severn Sound Environmental Association • Buffalo River and Niagara River: Buffalo Niagara Waterkeeper • Buffalo River and Niagara River: Buffalo Niagara Waterkeeper
	Fosters integration through broad-based institutional structure	
	Promotes a watershed focus	
	Builds collaboration, partnerships, and capacity	
Effective watershed management has been facilitated through strengthened connections to networks external to RAPs often through a local or regional organization	Promotes a watershed focus	• Buffalo River and Niagara River: Buffalo Niagara Waterkeeper • Saginaw River and Bay: Saginaw Bay Watershed Initiative Network • Rochester Embayment: Water Resources Board of the Finger Lakes – Lake Ontario Watershed Protection Alliance • Rouge River: Alliance of Rouge River Communities
	Builds collaboration, partnerships, and capacity	
Partnering with scientific institutions has strengthened science-policy-management linkages and can cultivate local scientific capacity	Connects to scientific organizations to understand problems and causes, and support scientifically-sound decision-making	• St. Lawrence at Cornwall: St. Lawrence River Institute of Environmental Science • Lakehead University’s InfoSuperior website for North shore AOCs
Institutional arrangements have grown from and evolved into a constellation of strategic partnerships	Builds collaboration, partnerships, and capacity	• Northshore AOCs partnered with Lake Superior Program office early on in RAP development and implementation • The Ashtabula River Partnership comprised of government and private entities to foster cooperative approach to sediment remediation
RAP institutional structures are increasingly looking beyond just restoring beneficial uses and addressing sustainability and life after delisting	Emphasizes sustainability planning for life after delisting as an AOC characteristics.	• The Muskegon Lake Watershed Partnership has developed a Muskegon Lake Ecosystem Action Plan to facilitate the continuation of coordinated, natural resources stewardship of the watershed for long-term sustainability [30]. • Muskegon Conservation District established environmental stewardship program and, following delisting, the White Lake Environmental Network focused on maintaining momentum for revitalization • Flats Forward is working with partners on community and economic development in the Flats of the Cuyahoga River AOC • Waterfront Toronto is working with partners to reconnect people to the Lake Ontario waterfront and achieve economic revitalization
Eight AOCs did not experience significant changes to their institutional arrangement likely due to original arrangements being well-suited to implement the RAP and/or the co-existing presence of a regulatory remediation program may have driven RAP progress without needing to amend its institutional arrangement.	Not applicable.	• Torch Lake • Deer Lake • Manistique River • Lower Menominee River • Waukegan Harbor • Wheatley Harbour • Oswego River • Port Hope
